# Draft Genome Sequence of the Phenazine-Producing Pseudomonas fluorescens Strain 2-79

**DOI:** 10.1128/genomeA.00130-15

**Published:** 2015-03-26

**Authors:** Kai Nesemann, Susanna A. Braus-Stromeyer, Andrea Thuermer, Rolf Daniel, Dmitri V. Mavrodi, Linda S. Thomashow, David M. Weller, Gerhard H. Braus

**Affiliations:** a Department of Molecular Microbiology and Genetics, Institute of Microbiology and Genetics, Georg-August Universität, Göttingen, Germany; b Department of Genomic and Applied Microbiology and Göttingen Genomics Laboratory, Institute of Microbiology and Genetics, Georg-August Universität, Göttingen, Germany; c Department of Biological Sciences, University of Southern Mississippi, Hattiesburg, Mississippi, USA; d USDA-ARS Root Disease and Biological Control Research Unit, Washington State University, Pullman, Washington, USA

## Abstract

Pseudomonas fluorescens strain 2-79, a natural isolate of the rhizosphere of wheat (Triticum aestivum L.), possesses antagonistic potential toward several fungal pathogens. We report the draft genome sequence of strain 2-79, which comprises 5,674 protein-coding sequences.

## GENOME ANNOUNCEMENT

The concentration and composition of antibiotic-producing, root-colonizing organisms are important factors that partially determine the suppressiveness of soils toward certain soil-borne diseases (1).

Fluorescent pseudomonads play a major role in suppressing take-all disease of wheat caused by the fungal pathogen Gaeumannomyces graminis var. *tritici* (Sacc.) (2). In 1979, Weller and Cook isolated bacteria from roots of wheat plants grown in take-all suppressive soils in Washington state, USA (3). Pseudomonas fluorescens 2-79 (NRRL B-15132) was characterized as a strong biological control agent suppressing G. graminis in vitro and *in planta*. Wheat plants infected with G. graminis var. *tritici* and additionally treated with P. fluorescens 2-79 resulted in taller plants, more heads, and fewer symptoms of root disease compared to the control plants without bacterial treatment. Bacterial treatment could increase the yield up to 147% in soils fumigated with methyl bromide and up to 27% in natural soils (3). P. fluorescens 2-79 produces phenazines, which represent a diverse chemical group of nitrogen-containing heterocyclic pigments possessing broadly inhibitory properties toward bacteria and fungi (4). Phenazines undergo redox reactions with NADH/NADPH, leading to an increase of toxic superoxide radicals and hydrogen peroxide in the target cells (5). Mavrodi et al. investigated the biosynthesis pathway of phenazines in P. fluorescens 2-79 (6).

Genomic DNA of P. fluorescens 2-79 was isolated by using the MasterPure Complete DNA and RNA purification kit (Epicentre, Madison, WI, USA). A shotgun sequencing library was generated employing the Nextera DNA sample preparation kit following the manufacturer’s instructions. The whole genome of P. fluorescens 2-79 was sequenced with the Genome Analyzer IIx (Illumina, San Diego, CA, USA). In total, 8.5 million paired-end reads of 112 bp were generated. *De novo* assembly of all shotgun reads using SPAdes version 3.0.0 (7) resulted in 143 contigs >3 kb and 123-fold coverage. The draft genome sequence comprises 6.4 Mb and a GC content of 59.83%. Genome annotation was performed by using Prokka (8). The draft genome harbored 1 rRNA cluster, 47 tRNA genes, 4,286 protein-encoding genes with function prediction, and 1,388 genes coding for hypothetical proteins.

Proteins involved in secondary metabolism were analyzed. The gene *hcnA* (GenBank accession no. 15560558) involved in HCN synthesis and the phenazine operon (GenBank accession no. L48616.1) are present in P. fluorescens 2-79. The gene *phlD* (GenBank accession no. 15563828) necessary for the synthesis of 2,4-diacetylphloroglucinol (DAPG) is absent in 2-79.

### Nucleotide sequence accession numbers.

This whole-genome shotgun project has been deposited at DDBJ/EMBL/GenBank under the accession number JXCQ00000000. The version described in this paper is the first version, JXCQ01000000.
